# Transcriptome Analyses in BV2 Microglial Cells Following Treatment With Amino-Terminal Fragments of Apolipoprotein E

**DOI:** 10.3389/fnagi.2020.00256

**Published:** 2020-08-13

**Authors:** Tanner B. Pollock, Giovan N. Cholico, Noail F. Isho, Ryan J. Day, Tarun Suresh, Erica S. Stewart, Madyson M. McCarthy, Troy T. Rohn

**Affiliations:** ^1^Department of Biological Sciences, Boise State University, Boise, ID, United States; ^2^Health Sciences Department, University of Washington School of Medicine, Seattle, WA, United States

**Keywords:** apolipoprotein E4, microglia cells, BV2 cells, Alzheimer’s disease, inflammation, toxicity, RNA-seq, M1 phenotype

## Abstract

Despite the fact that harboring the apolipoprotein E4 (*APOE4*) allele represents the single greatest risk factor for late-onset Alzheimer’s disease (AD), the exact mechanism by which ApoE4 contributes to disease progression remains unknown. Recently, we demonstrated that a 151 amino-terminal fragment of ApoE4 (nApoE4_1–151_) localizes within the nucleus of microglia in the human AD brain and traffics to the nucleus causing toxicity in BV2 microglia cells. In the present study, we examined in detail what genes may be affected following treatment by nApoE4_1–151_. Transcriptome analyses in BV2 microglial cells following sublethal treatment with nApoE4_1–151_ revealed the upregulation of almost 4,000 genes, with 20 of these genes upregulated 182- to 715-fold compared to untreated control cells. The majority of these 20 genes play a role in the immune response and polarization toward microglial M1 activation. As a control, an identical nApoE3_1–151_ fragment that differed by a single amino acid at position 112 (Cys→Arg) was tested and produced a similar albeit lower level of upregulation of an identical set of genes. In this manner, enriched pathways upregulated by nApoE3_1–151_ and nApoE4_1–151_ following exogenous treatment included Toll receptor signaling, chemokine/cytokine signaling and apoptosis signaling. There were unique genes differentially expressed by at least two-fold for either fragment. For nApoE3_1–151_, these included 16 times as many genes, many of which are involved in physiological functions within microglia. For nApoE4_1–151_, on the other hand the number genes uniquely upregulated was significantly lower, with many of the top upregulated genes having unknown functions. Taken together, our results suggest that while nApoE3_1–151_ may serve a more physiological role in microglia, nApoE4_1–151_ may activate genes that contribute to disease inflammation associated with AD. These data support the hypothesis that the link between harboring the *APOE4* allele and dementia risk could be enhanced inflammation through activation of microglia.

## Introduction

Alzheimer’s disease (AD) is the most common form of dementia and is classified as a progressive, neurodegenerative disease whose symptoms include loss of memory and higher executive functioning (DeTure and Dickson, 2019). A prominent factor for late-onset AD is apolipoprotein E (ApoE), a lipoprotein transporter encoded by a single gene with three alleles (*APOE2*, *E3*, and *E4*). The *APOE4* allele is found in approximately 40% of AD patients and represents the greatest genetic risk factor for late-onset AD (Raber et al., 2004). In contrast, harboring the *APOE3* allele is neutral in dementia risk even though this version differs by only a single amino acid from ApoE4, having a cysteine at position 112 instead of arginine (Raber et al., 2004). A central question is how does this single amino acid difference account for dementia risk on a molecular level?

Studies have suggested that the key could be enhanced proteolysis of ApoE4 into fragments that have a toxic-gain of function (Rohn, 2013). Thus, ApoE4 is proteolyzed more readily than ApoE3, and fragments of ApoE4 are more prevalent in the brains of AD patients (Huang et al., 2001; Rohn et al., 2012; Rohn, 2013). Recently, we extended these findings by demonstrating that an amino-terminal fragment of ApoE4 (nApoE4_1–151_) generated following cleavage of full-length ApoE4 by extracellular cellular proteases including MMP-9 is taken up by microglia, targets to the nucleus, and can induce cytotoxicity (Love et al., 2017). We also found the presence of this fragment in the nucleus of microglia in both *E4/E4* and *E3/E3* cases of postmortem AD brain sections (Love et al., 2017). Our hypothesis is that nApoE4_1–151_ acts as a transcription factor leading to the expression of genes that promote microglia activation (Pollock et al., 2019). In the present study we examined this possibility by utilizing BV2 microglia cells and demonstrated that sublethal concentrations of nApoE4_1–151_ promoted upregulation of thousands of genes, many linked to the functioning of the immune system and microglia activation. Surprisingly an identical nApoE3_1–151_ fragment (differing by one amino acid at position 112, C→R) also led to an upregulation of many of the same genes as nApoE4_1–151_. These results suggest a novel role for ApoE and further, potentially link harboring the *APOE4* allele to inflammation and degeneration that has long been associated with AD (Streit, 2005; Graeber and Streit, 2010; Heneka et al., 2014; Das and Chinnathambi, 2019).

## Materials and Methods

### Materials

Construction and purification of the amino-terminal fragments 1–151 for ApoE4 (nApoE4_1–151_) or ApoE3 (nApoE3_1–151_) was contracted out to GenScript (Piscataway, NJ, United States). For both fragments, a 6X-His tag was coupled to the fragments to facilitate purification. Mouse CXCL2 or IL-12β quantikine ELISA kits were purchased from R&D Systems (Minneapolis, MN, United States).

### Cell Culture of BV2 Cells

BV2, murine microglial cells, were maintained at 37°C and 6% CO_2_ in a humidified incubator. Cells were maintained in RPMI 1640 Media (Hyclone) supplemented with 10% standard fetal bovine serum (Hyclone), 10% Cellgro MEM Non-essential Amino Acid (Corning) and 10% Penicillin streptomycin (Hyclone). Cells were cultured in 50 mL T25 Flasks. All supplies were purchased from ThermoFisher Scientific Inc. (Waltham, MA, United States). Treatment of BV2 cells was undertaken by incubation with nApoE3_1–151_ or nApoE4_1–151_ fragments diluted in conditioned media at a concentration of 25 μg/ml for 5 h to assess mRNA expression. Control cells (untreated) had an equivalent amount of conditioned media added to the wells.

### Total RNA Extraction and cDNA Synthesis in BV2 Microglia Cells

Total RNA was extracted from cells with the Direct-zol RNA MicroPrep Kit (Zymo Research Corp., CA, United States) according to manufacturer’s instructions. Genomic DNA was eliminated using TURBO DNAse as described by the manufacturer (Life Technologies, CA, United States). RNA quality was assessed using spectrophotometry and gel electrophoresis. Total cDNA was generated from 1 (μg of total RNA using qScript cDNA SuperMix (QuantaBio, MA, United States). Prior to use in qPCR, cDNA was diluted 1:2 with water.

### CXCl2 and IL-12b Quantitative PCR

Primers were designed to specifically amplify a portion of either the IL-12β or CXCL2 genes. Serine/arginine-rich splicing factor 11 (SFRS11) and EH domain-binding protein 1 (EHBP), two ultraconserved elements that have invariant copy number in mice, were used as reference genes. All primers were synthesized by Integrated DNA Technologies (Coralville, IA). For *Il12*β, the forward sequence was TGGTTTGCCATCGTTTTGCTG and the reverse was ACAGGTGAGGTTCACTGTTTCT. For *CXCL2*, the forward sequence was CGCTGTCAATGCCTGAAGAC and the reverse was ACACTCAAGCTCTGGATGTTCTTG. Primer efficiencies (E%) were confirmed to be between 90 and 110%. Primers were confirmed to be specific based upon melting profiles:

**Table:** 

Gene	Forward	Reverse	E%
SFRS11	AAATACCACCCAAC AGTTT	AAGCCCTATACAGA TGGAT	101
EHBP	GAGTCTCCAATATCAT CAGTAAGC	ACACATGCCACGA TCAATG	96
CXCL2	CGCTGTCAATGCCT GAAGAC	ACACTCAAGCTCTGGAT GTTCTTG	101
IL12B	TGGTTTGCCATCGTTT TGCTG	ACAGGTGAGGTTCACTG TTTCT	99

The total volume for each reaction was 20 μl and included 10 μl Forget-Me-Not EvaGreen qPCR Master Mix (Biotium Inc., Ca, United States), 1 μl of each appropriate primer (10 μM), 4 μl of nuclease free water, and 4 μl of template cDNA. Each PCR reaction also included a reverse transcription negative control to confirm the absence of genomic DNA in triplicate and a non-template negative control to confirm the absence of primer dimerization in triplicate. Real-time qPCR was run on a LightCycler 96 (Roche, Basel, Switzerland). The cycling conditions were 1 cycle of denaturation at 95°C for 2 min, followed by 40 cycles of amplification (95°C for 5 s, 55°C for 10 s, and 72°C for 15 s) and one cycle of product melting (95°C for 10 s, 65°C for 60 s, and 97°C for 1 s). All samples were amplified in triplicate, and the Cq value for each reaction was determined by the LightCycler 96 SW1.1. Relative differences in expression between treatments were determined by the LightCycler 96 SW1.1 and confirmed with the ΔΔCt method.

### RNA-Sequencing, Mapping, and Analysis

#### RNA-Sequencing

RNA-sequencing was performed by the Molecular Research Core Facility at Idaho State University (Pocatello, ID). All samples were sequenced using an Illumina HiSeq4000 Sequencer. Reads of 1 × 75 bp were demultiplexed and adapter sequences were removed using Trim Galore v0.5.0^1^. Trimmed reads were then assessed for quality using FASTQC v0.11.8^2^. Reads were then mapped to a mouse reference genome (version GRCm38.p6) using Hisat2 v2.1.0 (Kim et al., 2015). Gene counts were determined using HTSeq v0.11.0 (Anders et al., 2015) after which, counts were normalized using the median-of-ratios method with Deseq2 v1.22.2 (Love et al., 2014). Deseq2 was then used to calculate *p*-values using a Wald test with a Benjamini-Hochberg *post hoc* correction. Genes with an adjusted *p*-value < 0.05 and a fold-change >2 were considered to be differentially expressed. Differentially expressed genes (DEGs) were enriched for gene ontologies (GO) using PANTHER^3^. Analyses were conducted to assess biological processes, molecular functions, and cellular components.

The data discussed in this publication have been deposited in NCBI’s Gene Expression Omnibus and are accessible through GEO Series accession number GSE153454^4^.

#### Gene Ontology Analysis

A list of every influenced gene and their respective fold changes was imputed into the PANTHER classification system (Shannon et al., 2003; Bindea et al., 2009). Statistical enrichment tests were performed against the musculus genome, and the Bonferroni correction was used. This process was performed for biological processes and pathways (Mi et al., 2019a), and the respective outputs were used. All data were statistically significant with a corrected *p*-value < 0.05. In addition, raw data was used to create a list of all genes upregulated by either nApoE3_1–151_ or nApoE4_1–151_ and a separate list of all genes downregulated by nApoE_1–151_ fragments was imputed into the PANTHER classification system (Mi and Thomas, 2009; Mi et al., 2019b) and a functional classification analysis was performed.

#### Statistical Analysis

Transcriptome data was used for analysis by averaging three independent measurements. Treatment group data was then referenced as a percent fold change increase from controls. Data was segregated and organized by gene and treatment group. The organization of data was to utilize the nApoE4_1–151_ (E4 group) as a standard, organizing the data by highest to lowest change for the E4 treatment group and aligning the fold change for the nApoE3_1–151_ (E3 group) to those values for a matched pairs system. Data was then pulled into the statistical analysis program, R, as a.csv file and checked for normality assumptions using a Shapiro-Wilks Normality test on each treatment group. Data failed to conform to normality. A Spearman Rank Correlation was used as opposed to Pearson’s Correlation due to the lack of normality in data and extreme values. The Spearman Rank Correlation was then run on the top 500 highest fold change genes for the E4 group in comparison to the matched genes in the E3 group to identify similarities in trends. The resulting rho value was then referenced alongside data to generate Figure 6.

### Confocal Microscopy

Following treatment studies, BV2 cells were fixed by incubating cells in 4% paraformaldehyde for 23 min. For antibody labeling, cells were washed with 0.1 M Tris–buffered saline (TBS), pH 7.4, and pretreated with 3% hydrogen peroxide in 10% methanol to block endogenous peroxidase activity. Slides were subsequently washed in TBS with 0.1% Triton X-100 (TBS-A) and then blocked for 30 min in TBS-A with 3% bovine serum albumin (TBS-B). Slides were further incubated overnight at room temperature with the anti-His rabbit antibody (1:5,000). Following two washes with TBS-A and a wash in TBS-B, slides were incubated with the anti-rabbit HRP-conjugated secondary antibody. Visualization was accomplished by using a tyramide signal amplification kit (Molecular Probes, Eugene, OR, United States) consisting of Alexa Fluor 488-labeled tyramide (green, Ex/Em = 495/519). Slides were mounted using ProLong Gold Antifade Mountant with DAPI (Molecular Probes). Images were taken with a Zeiss LSM 510 Meta system combined with the Zeiss Axiovert Observer Z1 inverted microscope and ZEN 2009 imaging software (Carl Zeiss, Inc., Thornwood, NY, United States). Confocal Z-stack and single plane images were acquired with an Argon (488 nm) and a HeNe (543 nm) laser source. Z-stack images were acquired utilizing the Plan-Apochromat 63 × /NA 1.4 and alpha Plan-Fluar 100 × /NA1.45 Oil objectives and with the diode (405 nm) and Argon (488 nm) laser sources, emission band passes of 505–550 nm for the detection of the nApoE1–151 (green channel, Alexa Fluor 488). Orthogonal projection images were constructed from Z-stacks in order to demonstrate the staining is nuclear.

## Results

### Confirmation of Nuclear Localization of Amino-Terminal Fragments of apoE in BV2 Microglial Cells

**FIGURE 1:**
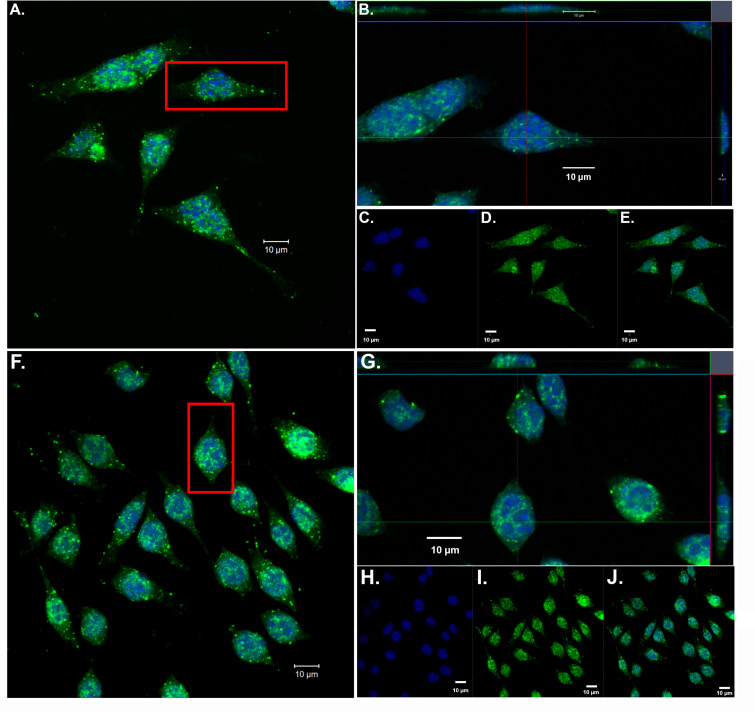
Orthogonal projections of confocal Z-stacks show nuclear localization of amino-terminal fragments of nApoE3 and E4 following exogenous treatment in BV2 microglia cells. BV2 microglia cells were treated with either 25 μg/ml of nApoE3_1–151_ **(A–E)** or with nApoE4_1–151_ **(F–J)** for 24 h, fixed, and immunostained with anti-His antibody to detect the localization of the apoE fragments. Confocal images were captured and Z-stacks were constructed showing the subcellular localization of nApoE fragments. **(A)** High magnification merged image depicting nApoE3_1–151_ in green and DAPI nuclear staining in blue. **(B)** Inset orthogonal projection from red rectangle in panel **A** demonstrating nuclear localization of nApoE3_1–151_ in a single BV2 microglia cell (crosshair). **(C–E)** Low magnification of nApoE3_1–151_ labeling with DAPI (blue, **C**), nApoE3_1–151_ (green, **D**), and the overlapped image **(E)**. All scale bars represent 10 μm. **(F–J):** Identical to panels **A–E** except staining is representative of nApoE4_1–151_ labeling. As with nApoE3_1–151_, orthogonal images clearly demonstrate the nuclear labeling of nApoE4_1–151_ (crosshair, **G**). Data are representative of five individual experiments.

Our previous findings have demonstrated the nuclear localization of amino-terminal fragments of apoE4 within microglia both *in vivo*, in the AD brain and *in vitro* following exogenous treatment of BV2 microglia cells (Love et al., 2017). To confirm and extend these findings, we exogenously treated BV2 microglia cells with both nApoE3_1–151_ and nApoE4_1–151_ fragments, which differ by a single amino acid (C→R). As shown in Figure 1, uptake and trafficking to the nucleus was apparent for both fragments, based on orthogonal projections taken from Z-stack images (Figure 1, crosshairs, panels B and G). These results are consistent with our previous findings (Love et al., 2017).

### Transcriptome Analysis of BV2 Microglia Cells Following Treatment With ApoE4_1–151_

**FIGURE 2:**
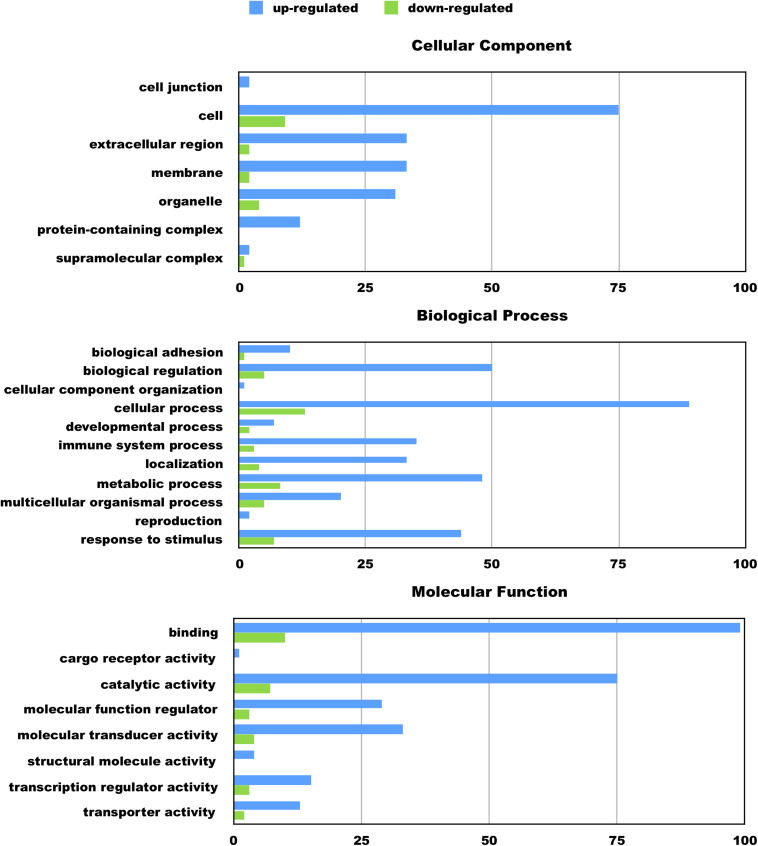
Functional categorization of up- and downregulated genes in BV2 microglia cells treated with an amino-terminal fragment of apoE4. BV2 cells were left untreated or treated for 5 h with nApoE4_1–151_ followed by total RNA purification and transcriptome analyses. The *x*-axis reflects the number of genes included for each component, process, or function. For example, there were a total of 75 upregulated genes categorized as being a part of the cellular component. Functional characterization was based on gene ontology (GO) annotations. Detailed information is shown in Supplementary Data File S1.

We hypothesize the nApoE4_1–151_ fragment is taken up by microglia cells, traffics to the nucleus and may act as a transcription factor leading to the change in gene expression. For example, we have recently demonstrated that treatment of BV2 cells with nApoE4_1–151_ led to an increase in the expression and release of the inflammatory cytokine, TNFα, a key trigger of microglia activation (Pollock et al., 2019). In addition, we demonstrated a specific binding interaction of nApoE4_1–151_ with the TNFα promoter region (Pollock et al., 2019). In the current study, to test whether nApoE4_1–151_ leads to the induction of a broader array of inflammatory genes or other pertinent genes, we performed a transcriptome analysis following treatment of BV2 microglia cells with a sublethal concentration of nApoE4_1–151._ In this regard, a concentration of 25 μg/ml showed no toxic effects following morphological examination and LDH measurements. Cells were viable, healthy, and displayed no indication of degeneration. LDH values for controls (relative units) were 0.34 ± 0.096 SEM and 0.370 ± 104 SEM for treated cells (*N* = 3, *p*-value = 0.80). In comparison to untreated control BV2 cells nearly 8000 genes were determined to be differentially expressed in the presence of nApoE4_1–151_. The raw data file can be accessed through Supplementary Data File S1. In addition, files containing the raw data are available in the GEO repository (GEO accession number, GSE153454). Although there were a similar number of up-regulated and down-regulated genes, the up-regulated genes had an average of a 6.6-fold change and the down-regulated genes had an average of a 2.2-fold change. Of the twenty most up-regulated genes, thirteen are known to be involved in the inflammatory immune response (Table 1). Biological processes, cellular components, molecular functions, and pathways for each gene were assigned by the PANTHER classification system (Figure 2). Involved cellular components included the cell, the extracellular region, the membrane etc. For each cellular component, there were many more associated genes that were upregulated than downregulated. The biological processes associated with genes differentially expressed in the presence of nApoE4_1–151_ included “cellular processes,” “biological regulation,” “response to stimulus,” and “immune system processes.” Each given biological process contained more up-regulated genes than down-regulated genes, with “cellular component organization” and “reproduction” being the least up-regulated.

**TABLE 1:** The twenty most up-regulated genes and their functions following treatment of BV2 microglia cells with nApoE4_1–151_.

Gene symbol	Gene name	Fold change	*p*-value	Function
Il12b	Interleukin 12B	715	1.59E-89	Cytokine
Gm41236	Predicted gene, 41236	692	2.50E-14	lncRNA located near Acod1
Gbp5	Guanylate binding protein 5	672	3.79E-149	Innate immune response
Cxcl2	C-X-C Motif chemokine ligand 2	505	0	Chemokine, may suppress cell proliferation
IFI44	Interferon induced protein 44	479	1.48E-12	Immune response
Hc	Hemolytic complement	459	3.93E-31	Innate immune response
Gm41236	Predicted Gene, 41236	337	5.74E-22	lncRNA located near Il7 gene
Acod1	Aconitate decarboxylase 1	322	0	Innate immune response
Adora2a	Adenosine A2a receptor	277	3.66E-27	Adenosine signaling
1700025C18Rik	RIKEN cDNA 1700025C18 gene	268	5.57E-10	lncRNA located near Cd40 gene
Lad1	Ladinin 1	262	4.94E-10	Cell structure
Gm41647	Predicted gene, 41647	225	2.34E-09	lncRNA near socs5 gene
Serpinb2	Serpin family B member 2	219	3.12E-09	Immune response
Il1b	Interleukin 1 beta	216	0	Cytokine
Zbp1	Z-DNA Binding protein 1	213	1.46E-18	Interferon production
Gbp2	Guanylate binding protein 2	213	0	Innate immune response
LOC105245043	Uncharacterized LOC105245043	199	2.97E-09	Uncharacterized
Traf1	TNF Receptor associated Factor 1	187	0	TNF signal transduction
Il27	Interleukin 27	183	7.26E-71	Cytokine
Cd69	Cd69 Molecule	182	7.75E-50	Immune response

*Of the known genes, >80% are associated with the immune system.*

**FIGURE 3:**
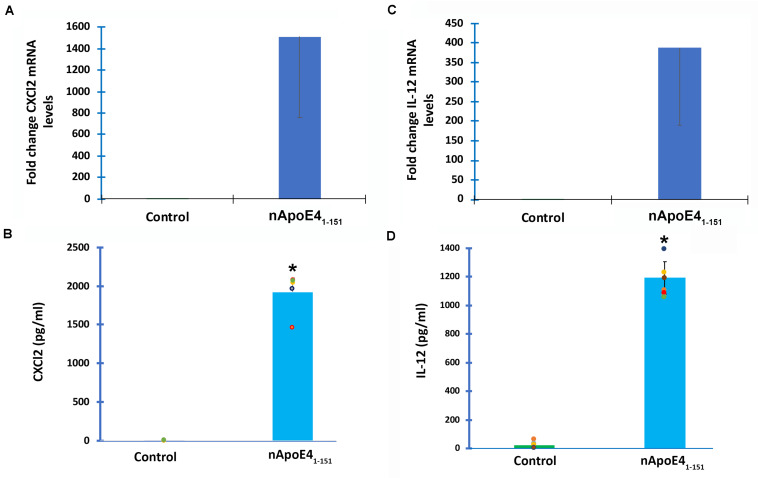
Validation of transcriptome analysis by RT-PCR and ELISA of two identified upregulated genes. Two genes (*CXCL2* and *IL12B*) identified by transcriptome analyses to be significantly upregulated in BV2 cells following treatment with 25 μg/ml apoE4_1–151_ (Table 1) were independently tested by RT-PCR **(A,C)** or ELISA **(B,D)** in order to validate transcriptome findings. **(A)** BV2 cells were left untreated (control, green bar) or treated for 5 h with 25 μg/ml nApoE4_1–151_ (blue bar), and RNA was extracted. RT-PCR analysis indicated a ∼1,500-fold increase in the expression of the inflammatory chemokine, CXCl2. Data are representative of two independent experiments. **(B)** Secreted CXCl2 levels are significantly elevated following treatment of BV2 cells with nApoE4_1–151_ (blue bar) as compared to untreated controls (green bar). Data are representative of five independent experiments ± SEM *denotes *p*-value is <0.0001 between control and nApoE4_1–151_. **(C)** BV2 cells were left untreated (control, green bar) or treated for 5 h with 25 μg/ml nApoE4_1–151_ (blue bar), and RNA was extracted. RT-PCR analysis indicated a ∼388-fold increase in the expression of the inflammatory cytokine, IL-12b. Data are representative of two independent experiments. **(D)** Secreted IL-12b levels are significantly elevated following treatment of BV2 cells with nApoE4_1–151_ (blue bar) as compared to untreated controls (green bar). Data are representative of eight independent experiments ± SEM *denotes *p*-value is < 0.00001 between control and nApoE4_1–151_.

To verify transcriptome results, two genes were chosen and independently verified by both RT-qPCR and ELISA assays. In comparison to untreated controls, the differential expression of two genes, *CXCL2* and *IL-12B*, and their corresponding proteins were confirmed with qPCR and ELISA (Figure 3). In this regard following treatment of BV2 microglia cells with nApoE4_1–151_, both *CXCL2* and *IL-12B* showed large increases in the fold mRNA levels (Figures 3A,C) as well as in the secreted protein in conditioned media (Figures 3B,D).

**FIGURE 4:**
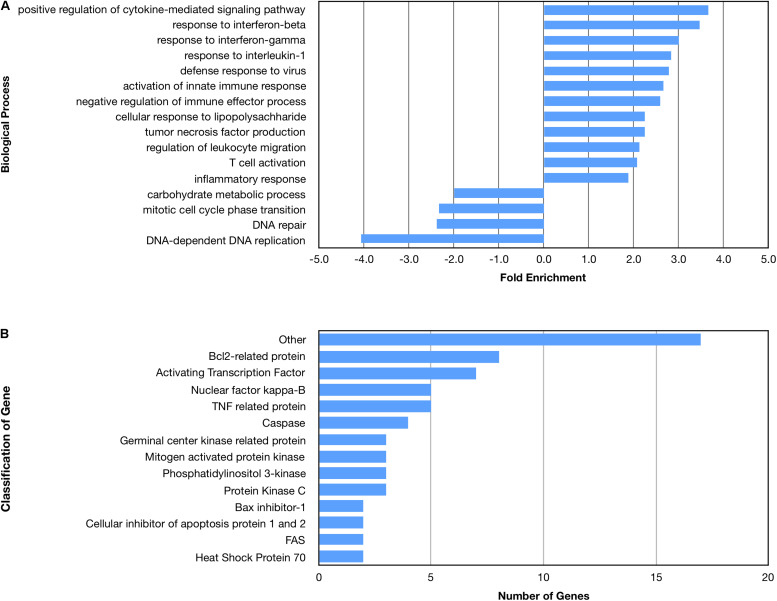
Enriched biological processes and classification of apoptosis related genes regulated by an amino terminal fragment of nApoE4. BV2 cells were plated onto 6-well plates to confluency and treated with or without ApoE4_1–151_ for 5 h. Following treatment, total RNA was extracted and transcriptome analysis was carried out as described in the Materials and Methods. **(A)** Data are expressed as fold enrichment of biological processes in BV2 microglia cells in the presence of the ApoE4_1–151_. Up-regulated processes are involved in the inflammatory immune response and the activation of BV2 microglia, while down-regulated processes are involved in cell division. **(B)** Classification of genes in the apoptosis signaling pathway regulated by ApoE4_1–151_. A total of 66 out of 72 genes in the apoptosis signaling pathway (P00006) were influenced by the introduction of ApoE4_1–151_. Enrichment and classification analyses were conducted using the PANTHER classification system (pantherdb.org).

Many biological processes involved in the inflammatory immune response were enriched by the nApoE4_1–151_ fragment, while many processes involved in cell division were down regulated (Figure 4A). The molecular functions of regulated genes included binding, catalytic activity and molecular transducer activity. Each molecular function contained more related up-regulated genes than down-regulated genes. Numerous pathways related to the inflammatory immune response were also enriched, including the apoptosis signaling pathway (Figure 4B). Of the 72 genes in the apoptosis signaling pathway (P00006), 66 were differentially expressed following the introduction of nApoE4_1–151_.

### Transcriptome Analysis of BV2 Microglia Cells Following Treatment With ApoE3_1–151_

We have previously documented the nuclear presence of nApoE_1–151_ fragments within microglia of the human AD brain whose genotype was *E3/E3* (Love et al., 2017). This would suggest that nApoE3_1–151_ may also act as a transcription factor leading to changes in gene expression. Therefore, we also performed an identical transcriptome analysis following treatment of BV2 microglia cells with nApoE3_1–151_. It is important to note, that in our previous study, treatment of nApoE3_1–151_ did not lead to cell toxicity (Love et al., 2017). As an initial approach, a broad overview of the similarities and differences between the two fragments was investigated. Treatment with nApoE3_1–151_ led to a differential upregulation of 2,262 genes by at least two-fold as compared to 1,617 genes for nApoE4_1–151_. Examining the top 20 genes that were induced by nApoE3_1–151_, the representative fold change ranged from 118- to 410-fold increased as compared to non-treated control cells (Table 2). This is in contrast to a representative fold change of 182- to 714-fold increase following treatment by nApoE4_1–151_ (Table 1). Of these 20 genes, 7 were also in the top 20 for nApoE4_1–151_-induced genes including *II12B*, *GM41236*, *GBP5*, *CXCl2*, *ACOD1*, *II1B*, and *GBP2*. Of the known top 20 genes, 70% are known to be associated with immune function (Table 2).

**TABLE 2:** The twenty most up-regulated genes and their functions following treatment of BV2 microglia cells with nApoE3_1–151_.

Gene symbol	Gene name	Fold change	*p*-value	Function
Gm41236	Predicted gene, 41236	410	7.59E-17	lncRNA located near Acod1
Cxcl2	C-X-C Motif chemokine Ligand 2	293	0	Chemokine, may suppress cell proliferation
Nos2	Nitric oxide synthase 2	290	1.10E-180	Produces nitric oxide
Gbp5	Guanylate binding protein 5	279	2.64E-198	Innate immune response
Csf3	Colony stimulating factor 3	224	9.65E-126	Cytokine
Il12b	Interleukin 12B	221	2.56E-88	Cytokine
Gm33055	Predicted gene, 33055	201	4.22E-09	lncRNA with uncharacterized function
Acod1	Aconitate decarboxylase 1	191	0	Innate immune response
Il1b	Interleukin 1 beta	180	9.60E-159	Cytokine
Gbp6	Guanylate binding protein 6	169	4.28E-41	Innate immune response
Gbp2	Guanylate binding protein 2	164	0	Innate immune response
II1f9	Interleukin 1F9	163	8.62E-12	Cytokine
Rsad2	Radical S-adenosyl methionine domain-containing protein 2	153	1.41E-93	Innate immune response
Ifi205	Interferon activated gene 205	149	1.13E-124	Innate immune response
Rnase10	RNase A family, 10	144	1.02E-07	Ribonuclease activity
Gm4951	Predicted gene, 4951	132	2.86E-20	Response to cytokine
Vcam1	Vascular cell adhesion molecule 1	130	6.19E-138	Immune response
Zfp811	Zinc Finger protein 811	122	1.72E-59	Nucleic acid binding
Kcnf1	Potassium voltage-gated channel modifier subfamily F member 1	118	6.50E-07	Putative voltage-gated potassium channel
Mefv	MEFV Innate immunity regulator	118	6.56E-07	Innate immune response

*Of the known genes, 70% are associated with the immune system.*

**FIGURE 5:**
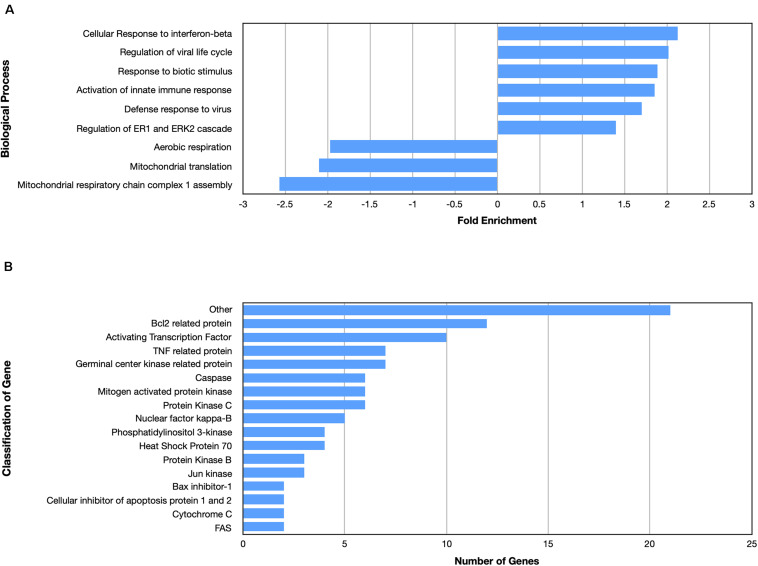
Enriched biological processes and classification of apoptosis related genes regulated by an amino terminal fragment of nApoE3. BV2 cells were plated onto 6-well plates to confluency and treated with or without nApoE3_1–151_ for 5 h. Following treatment, total RNA was extracted and transcriptome analyses was carried out as described in the Materials and Methods. **(A)** Data are expressed as fold enrichment of biological processes in BV2 microglia cells in the presence of the nApoE3_1–151_. Up-regulated processes are involved in the inflammatory immune response of BV2 microglia, while down-regulated processes are involved in mitochondrial oxidative phosphorylation. **(B)** Classification of genes in the apoptosis signaling pathway regulated by nApoE3_1–151_. Numerous genes involved in cell signaling pathways were upregulated following treatment with the E3 fragment. Enrichment and classification analyses were conducted using the PANTHER classification system (pantherdb.org).

Examining the enriched upregulated pathways, many of those pathways are linked to the immune system, similar to what was found for the nApoE4_1–151_ fragment (Figure 5A). These enriched upregulated pathways included cellular responses to interferon-beta, regulation of viral life cycle, activation of the innate immune response and defense to viruses (Figure 5A). Our previous findings documented the toxicity of the nApoE4_1–151_ fragment, while the nApoE3_1–151_ showed no toxicity under identical treatment conditions. Therefore, like for the nApoE4_1–151_ (Figure 3B), we examined whether any genes related to apoptosis were upregulated. As shown in Figure 5B, there was a mix of anti-apoptotic genes induced (Bcl-2 related), Bax inhibitor-1, and cellular inhibitors of apoptosis of protein 1 and 2 as well as pro-apoptotic genes including TNF related protein, caspases, and cytochrome c (Figure 4B). Comparing the fold enrichment between the two fragments, there was a 3.66-fold increase in apoptosis signaling for nApoE4_1–151_ fragment versus 2.68 for the nApoE3_1–151_ (Table 3). However, these data cannot explain the difference in our previously observed toxicity between the two fragments (Love et al., 2017).

**TABLE 3:** Enriched pathways upregulated by nApoE3_1–151_ and nApoE4_1–151_.

Pathway	Enrichment by E3 fragment	Enrichment by E4 fragment
Toll receptor signaling	5.22	6.5
Chemokine/cytokine signaling	3.3	3.38
Apoptosis signaling	2.68	3.66
Integrin signaling		2.6

We also found similar fold increases in enrichment pathways for both fragments with regards to Toll receptor signaling and inflammation mediated by chemokine and cytokine signaling. The only difference was that for integrin signaling pathways, which was found to be specifically enriched (2.6-fold) for only the nApoE4_1–151_ fragment (Table 3).

Looking at gene pathways downregulated, there was a significant difference found between the two fragments. The nApoE3_1–151_ led to the down regulation of genes related to aerobic respiration, mitochondrial translation, and in mitochondrial respiratory chain complex 1 assembly (Figure 5A). In contrast, many processes involved in cell division were downregulated by the nApoE4_1–151_ fragment (Figure 4A).

**FIGURE 6:**
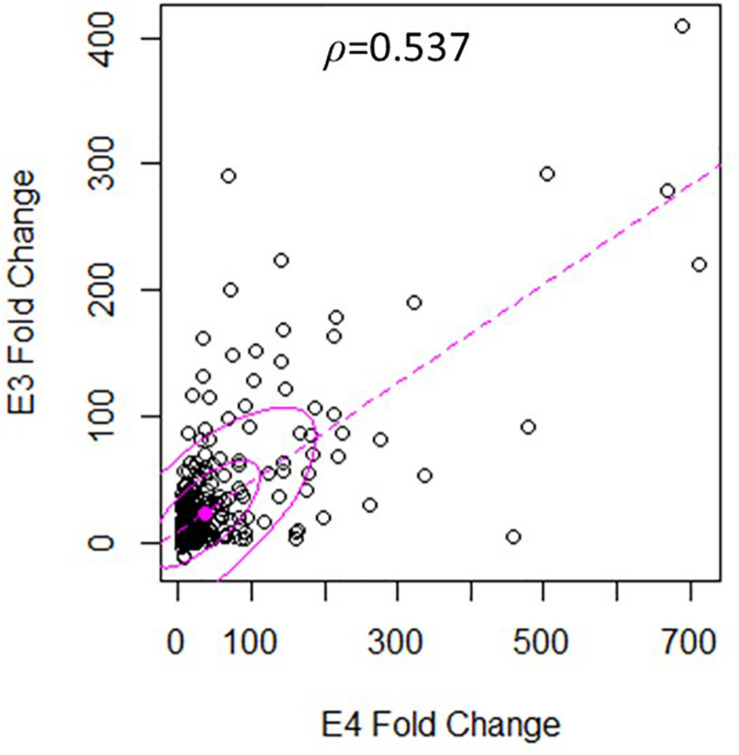
Gene expression following treatment with fragments displays similar patterns of upregulation. The scatterplot analysis displays a moderate positive relationship between the nApoE3_1–151_ and nApoE4_1–151_ treatment groups (*S* = 9651720, ρ = 0.537, *p*-value < 0.00). Data is representative of three independent trials that were averaged and expressed as a percentage change in comparison to the control versus each treatment. A Spearman Rank Correlation was utilized to overcome dissonance in normality from extreme data points. Ellipses represent 0.5 and 0.95 confidence intervals and the dotted line is a linear trendline.

There was a significant degree of overlap in the genes differentially expressed by both nApoE fragments. Following a correlation analysis with a pool of the top 500 upregulated nApoE4_1–151_ and nApoE3_1–151_ genes, the trajectories of the fold changes were similar for many of the genes (*S* = 9651720, ρ = 0.537, *p*-value < 0.00). These results indicate that while the degree of change may not always align for the two treatment groups, the direction of impact follows a weak positive correlation path (Figure 6). In most cases, even when genes were impacted in a similar fashion, the percent fold change in reference to the control was much higher for the nApoE4_1–151_ fragment than for the nApoE3_1–151_ fragment (Table 4).

**TABLE 4:** The top 15 overlapping genes upregulated by nApoE3_1–151_ and nApoE4_1–151_.

Gene symbol	E3 Fold change	E4 Fold change
Il12b	221	715
Gm41236	410	691
Gbp5	279	672
CXCl2	293	505
Ifi44	92	479
Hc	5.36	459
LOC102634900	53.2	337
Acod1	191	322
Adora2a	82.7	277
Lad1	30.2	262
Gm41647	86.3	225
Serpinb2	68.1	219
Il1b	180	216
Zbp1	102	213
Gbp2	164	213

### Unique Genes Differentially Regulated Following Treatment of BV2 Microglia Cells With Either nApoE3_1–151_ or nApoE4_1–151_

**FIGURE 7:**
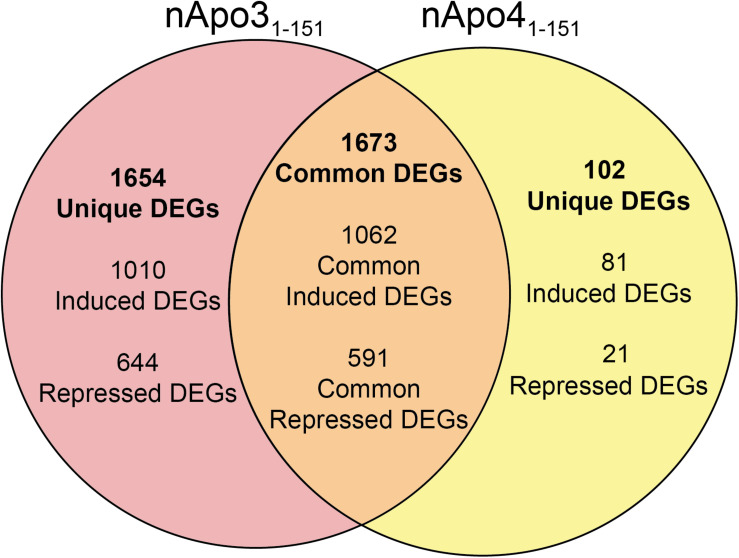
Venn diagram demonstrating common and divergent gene regulation between nApoE3_1–151_ and nApoE4_1–151_ fragments. A comparison of genes regulated more than two-fold by treatment with nApoE3_1–151_ and nApoE4_1–151_. nApoE3_1–151_ regulated the expression of 1654 genes that were not regulated by nApoE4_1–151_. Of these 1654 genes, 1010 were upregulated and 644 were downregulated. Conversely, there were 102 genes uniquely regulated by nApoE4_1–51_. Of these 102 genes, 81 were upregulated, and 21 were downregulated. There were 1673 genes that were regulated by both nApoE4_1–151_ and nApoE3_1–151_. Also included are common differentially expressed genes (DEGs) for both fragments including 1,062 induced DEGs and 591 repressed DEGs (orange intersecting region).

For the nApoE3_1–151_ fragment, using a minimum of two-fold increase/decrease change as our criteria, a total of 1,010 genes were upregulated by this fragment alone and 644 genes downregulated (Figure 7). Examining the putative functions of the top 10 unique upregulated genes, there was a diverse array of genes involved in cellular processes including, mitosis, regulation of the cytoskeleton, and cell signaling. The specific proteins expressed included those for a dopamine receptor (*DRD3* gene), potassium channel (*KCNF1* gene), collagen (*COL6A1* gene), G-protein signaling (*HCAR1* gene), and actin stability (*NES* and *XIRP1* genes).

Using the same criteria of at least a two-fold increase/decrease change in gene expression, a total of 81 genes were upregulated by the nApoE4_1–151_ fragment (Figure 7). For those genes downregulated following treatment with nApoE4_1–151_, a total of 21 genes were identified (Figure 7). Table 5 depicts the top 20 genes either upregulated or downregulated following treatment of BV2 microglia cells with the nApoE4_1–151_ fragment. In both cases, these changes represent significantly fewer numbers of genes differentially expressed as compared to nApoE3_1–151_. For the upregulated genes, examining the top twenty genes, only 6 (*ETNK2*, *H2-Q6*, *EVA1B*, *NAT8*, and *KCNQ2*) have known functions. The top five genes (*1700025C18Rik*, *1700003M07Rik*, *GM32846*, *GM36778*, *G930009F23RIK*) representing a fold change of 43 to 268-fold), have no known function. Other genes in this top 20 list have been associated with a number of different diseases including cancer (*SRMS*), epilepsy (*KCNQ2*), frontotemporal dementia (*TMEM252*), and amyotrophic lateral sclerosis (*TMEM252*).

**TABLE 5:** Top 20 genes differentially regulated by the nApoE4_1–151_ fragment.

Genes upregulated	Fold change	Genes downregulated	Fold change
1700025C18Rik	268	Adamtsl2	−38.0
1700003M07Rik	146	Treml1	−24.9
Gm32846	62.3	Zdhhc8	−20.1
Gm36778	42.7	Gm34741	−19.9
G930009F23Rik	38.9	Gm30624	−19.6
Etnk2	36.8	Gm38809	−18.4
H2-Q6	35.5	BE949265	−10.1
Eva1b	35.3	Gm26808	−9.35
Nat8	33.2	Gm34515	−7.94
Gm8075	31.5	Fam19a5	−7.61
Tmem252	31.3	Gm6416	−7.03
4930524O08Rik	29.6	Igdcc4	−6.51
LOC108168675	29.2	Pxt1	−5.61
Srms	28.0	4833415N18Rik	−5.30
Kcnq2	27.8	Pnpla5	−5.24
LOC100862202	27.6	LOC102632901	−4.63
LOC108167381	25.7	Mcidas	−3.82
Gm34542	25.6	Tpbgl	−3.21
Gm26509	25.4	Apbb1	−2.87
A530046M15Rik	25.3	Shc2	−2.72

## Discussion

ApoE is polymorphic with three major isoforms, ApoE2, ApoE3, and ApoE4, which differ by single amino acid substitutions involving cysteine-arginine replacements at positions 112 and 158 (Weisgraber et al., 1981). Harboring the *APOE3* allele is believed to neither increase nor decrease one’s risk of AD, whereas inheritance of the *APOE4* allele increases disease risk upward to tenfold (Eisenstein, 2011). It is noteworthy that 65–80% of all AD patients have at least one *APOE4* allele (Saunders et al., 1993; Farrer et al., 1997). The classic function of ApoE resides in the CNS, where it is produced by a variety of cells including microglia, and transports cholesterol to neurons via apoE receptors, which are members of the low-density lipoprotein (LDL) receptor family (Pitas et al., 1987; Michikawa et al., 2000). Although ApoE3 and ApoE4 differ by a single amino acid at position 112, none of the known actions of ApoE4 sufficiently explain how harboring this allele enhances AD risk. Recent evidence from our group has suggested that this single amino acid change leads to enhanced proteolysis of the full-length protein in several fragments, including a 151 amino-terminal fragment of ApoE4 (nApoE4_1–151_). We have demonstrated that this fragment is present in the human AD brain where it localizes to nuclei of microglia cells (Love et al., 2017). In addition, nApoE4_1–151_ is toxic *in vitro* in BV2 microglia cells and may induce toxicity by leading to the expression of inflammatory genes including TNFɑ (Love et al., 2017; Pollock et al., 2019). Moreover, nApoE4_1–151_ binds directly to the promoter region of TNFɑ and can induce expression and release of the TNFɑ following treatment of BV2 microglia cells (Pollock et al., 2019). The purpose of the present study was to determine in greater detail the ability of nApoE4_1–151_ to induce changes in gene expression following treatment of BV2 microglia cells. As a control, we directly compared transcriptome results with an identical nApoE3_1–151_ that differs by a single amino acid at position 112 (C > R). It is noteworthy that we could not test the role of an ApoE2 fragment in terms of changes in gene expression because a 1-151 amino-terminal fragment of ApoE2 would be identical to the nApoE3_1–151_ fragment. Bearing this in mind, it is difficult for us to speculate on what potential structural effects of ApoE2 could potentially modulate gene expression. However, it is possible that amino-terminal ApoE2 fragments may also have an effect on gene expression, but further studies will be necessary to address this issue.

Treatment of BV2 microglia cells with nApoE4_1–151_ led to the differential expression of a vast array of thousands of genes including a 715-fold increase in the expression of *IL12B* gene as well as a 504-fold increase in the *CXCL1* gene, both key mediators of inflammatory immune responses. We independently confirmed the increase in gene expression and protein secretion of IL12b, CxCl1 (Figure 3), and TNFɑ (Pollock et al., 2019) in BV2 cells by RT-PCR and ELISA assays. Overall, many biological processes involved in the inflammatory immune response were enriched by the fragment, while many processes involved in cell division were down regulated. In addition, apoptotic pathways were enriched by the nApoE4_1–151_ fragment providing a possible link to the observed toxicity *in vitro* (Love et al., 2017).

Examining the top five genes induced by nApoE4_1–151_ and their possible connection to AD, the top gene was *IL12B*, which increased 715-fold following treatment of BV2 microglia cells with nApoE4_1–151_. This cytokine has been implicated in the AD process. Elevated levels of the p40 subunit of IL-12 have been detected in AD brains (Pitas et al., 1987), and in AD animal models, inhibition of p40 alleviates the cognitive impairments and AD-related pathology (Vom Berg et al., 2012; Tan et al., 2014). Rs568408 and rs3212227 SNPs, which are located in *IL-12A* and *IL-12B*, respectively, have recently been reported to influence AD risk in the Han Chinese population (Zhu et al., 2014). The *GBP5* gene increased 672-fold following treatment with nApoE4_1–151_ and this gene belongs to the TRAFAC class dynamin-like GTPase superfamily. The encoded protein acts as an activator of NLRP3 inflammasome assembly and has a role in innate immunity and inflammation (Latz et al., 2013). The NLRP3 inflammasome is an important contributor to inflammatory diseases, including AD (Latz et al., 2013; Heneka, 2017; Ising et al., 2019). Finally, the *CXCl2* gene expression increased 505-fold following treatment. This gene is part of a chemokine superfamily that encodes secreted proteins involved in immunoregulatory and inflammatory processes. The CXCl2 protein and its receptor has been identified in AD patient brains and may promote Aβ-stimulated microglia activation (Boddeke et al., 1999; Van Coillie et al., 1999; El Khoury et al., 2003).

In summary, exogenous treatment of BV2 microglia cells with nApoE4_1–151_ led to the upregulation of thousands of genes and of the top 20, 80% are associated with microglia activation and inflammation. These data support a possible linkage between harboring the *APOE4* allele and inflammation that has long been associated with AD (Streit, 2005; Graeber and Streit, 2010; Heneka et al., 2014; Das and Chinnathambi, 2019).

We compared the transcriptome results of nApoE4_1–151_ with nApoE3_1–151_ which differ by a single amino acid at position 112. It is noteworthy that in a previous study, we identified nApoE3_1–151_ in postmortem human AD sections, although to a lower extent as to nApoE4_1–151_ (Love et al., 2017). In BV2 cells, although nApoE3_1–151_ appears to somewhat traffic to the nucleus, it does not lead to cell toxicity nor does it lead to a change of morphology indicative of activation (Love et al., 2017; Pollock et al., 2019). Therefore, we were somewhat surprised at the number of genes differentially regulated by the nApoE3_1–151_ fragment. Of the top twenty genes induced, 70% are involved in immune function. In addition, six of the top twenty genes upregulated were listed in the top twenty genes induced by the nApoE4_1–151_ fragment (Tables 1, 2). Like nApoE4_1–151_, nApoE3_1–151_ led to a 2.68-fold enrichment in apoptotic pathways, compared to 3.66-fold for nApoE4_1–151_ (Table 3). Therefore, at this time we cannot reconcile the lack of cell death as caused by treatment with nApoE3_1–151_ in BV2 cells (Love et al., 2017) and the upregulation of apoptosis signaling pathways shown in the present study. It is also important to note that our read depth was sufficient so that results are not biased, meaning that the differences in the significant genes that appear in one treatment versus another are not due to a difference in coverage, but that they are actually significant.

A significant difference between the two fragments was that only nApoE4_1–151_ led to enrichment in integrin signaling pathways (2.6-fold). Another interesting difference was the finding that the nApoE3_1–151_ uniquely led to the downregulation of pathways involved in mitochondrial function including aerobic respiration, mitochondrial translation and mitochondrial respiratory chain complex 1 assembly (Figure 5). Multiple lines of evidence suggest that mitochondrial integrity and function, and innate immunity are closely interlinked processes. Mitochondria are intracellular organelles required for numerous cellular functions including energy metabolism, regulation of reactive oxygen species (ROS) signaling, Ca^2+^ homeostasis, and apoptosis (Culmsee et al., 2018). Therefore, a potential consequence of down regulating genes associated with mitochondrial function may be a dampening of the production of ROS and an overall effect of moving microglia away from activation.

We also examined those genes whose expression changed with the treatment of nApoE3_1–151_ but did not change with the treatment of nApoE4_1–151_ and vice versa. Using a criterion of at least a two-fold change in expression, there were ∼16 times more genes differentially upregulated by nApoE3_1–151_ (1,010 genes) in comparison to nApoE4_1–151_ (81 genes). Many of these nApoE3_1–151_-induced genes appeared to be involved in an array of microglia processes including regulation of the cytoskeleton, cell signaling, and extracellular matrix pathways. In addition, one gene of interest was uniquely downregulated by nApoE3_1–151_ by ∼20-fold. This gene, *SLC25A11* encodes for mitochondrial, 2-oxoglutarate/malate carrier (Monne et al., 2013). A recent study demonstrated that this carrier plays a key role in regulation of the NLRP3 inflammasome, a multiprotein complex, which is involved in a pro-inflammatory form of cell death (Shuvarikov et al., 2018). Thus, the downregulation of SLC25A11 protein may prevent activation of this inflammasome and thus limit this form of cell death as well as subsequent inflammation.

In contrast to those genes specifically regulated by nApoE3_1–151_, many of the genes differentially expressed by nApoE4_1–151_ have unknown functions. For example, of the 20 top upregulated genes, only 6 (*ETNK2*, *H2-Q6*, *EVA1B*, *NAT8*, and *KCNQ2*) have known functions. The top five genes (*1700025C18RIK*, *1700003M07Rik*, *GM32846*, *GM36778*, *G930009F23RIK*) representing a 43-268-fold change), have no known function. Although no known function has been assigned to any of these top 5 genes, one gene where there is existing evidence is *1700025C18RIK*, which increased 146-fold following nApoE4_1–151_ treatment. *1700003M07Rik* encodes for AK005651 mRNA and exhibits decreased expression in thymus and spleen in non-obese diabetic mice. A type 1 diabetes susceptibility locus, *IDD11*, and recombination hotspot were found within this gene. As a result, this gene may play a role in diabetes (Tan et al., 2010; Hamilton-Williams et al., 2013). Limited data is also available on another relatively unknown gene, *G930009F23RIK* upregulated 39-fold by the nApoE4_1–151_ fragment. *G930009F23RIK* is transcribed to AK145170 lncRNA. Previous studies have shown AK145170 is upregulated 21-fold following treatment with negative factor (Nef) in mouse astrocytes (Zhou et al., 2018). Nef is a myristoylated, HIV-encoded protein released through exosomes that induces the production of various cytokines and chemokines in astrocytes to promote neuron death (van Marle et al., 2004). The effects of HIV-1 infection, especially through the neurotoxicity of Nef, may contribute to HIV-associated neurocognitive disorders (HAND) pathogenesis (Torres-Munoz et al., 2001). Thus, *G930009F23RIK* expression may play a role in the pathogenesis of HAND.

It is interesting to speculate on what role these top five genes may have and if they contribute somehow to the toxicity produced by nApoE4_1–151_, which was not observed with nApoE3_1–151_ (Love et al., 2017). Due to the paucity of unique genes induced by nApoE4_1–151_, we were unable to analyze any enriched pathways, however, the data did reveal two genes involved in the AD-presenilin pathway and one gene involved in the AD-Aβ secretase pathway. On the other hand, enriched pathways for nApoE3_1–151_, were easily calculated given the large number of genes upregulated and included collagen biosynthesis and modifying enzymes (5.17-fold enrichment), collagen formation (4.34-fold enrichment), class A/1 Rhodopsin-like receptors (2.54-fold enrichment), and GPCR ligand binding (2.23-fold enrichment). Taken together, these results can be interpreted to suggest that nApoE3_1–151_ may play a more physiological role in microglia cells as compared to nApoE4_1–151_, which may lead to activation, inflammation, and activation of AD pathways.

## Conclusion

Herein, we provide transcriptome data supporting a new role for ApoE3 and E4 as regulators of gene expression. Traditionally, ApoE has an important role in regulating the metabolism of lipids by directing their transport, delivery, and distribution from one cell type to another through ApoE receptors within the CNS (Verghese et al., 2011). Our previous findings supported a role for amino-terminal fragments of ApoE4 consisting of 1–151 amino acids (nApoE4_1–151_) that can traffic to the nucleus and bind to enhancer elements such as for TNFα, leading to expression, activation and cytotoxicity of BV2 microglia cells (Love et al., 2017). Full-length ApoE4 was without effect in this respect, and treatment of nApoE3_1–151_ did not lead to cellular toxicity. In the current study, we now extend these findings and demonstrate nApoE4_1–151_ and nApoE3_1–151_ can lead to large changes in gene expression following exogenous treatment of BV2 microglial cells. Our results suggest that while nApoE3_1–151_ may serve a more physiological role in this manner, nApoE4_1–151_ may activate genes with a more pathological purpose. These data support the hypothesis that the link between harboring the *APOE4* allele and dementia risk could be enhanced inflammation through activation of microglia to the inflammatory M1 phenotype (see below). Future studies are warranted to understand the set of genes uniquely upregulated by the nApoE_1–151_ fragment, many of which have an unknown function at this time. BV2 cells are a well-characterized, extensively employed model system for microglia. Studies have demonstrated that BV2 cells are a valid substitute for primary microglia in many experimental settings, including complex cell-cell interaction studies (Henn et al., 2009). However, there are clear limitations using these cells including that they are murine in origin and represent transformed cells, which could change their phenotype (Timmerman et al., 2018). Therefore, further studies will be needed in human iPSC-induced microglia cells or other primary cultures of microglia to confirm that similar sets of genes are expressed in human microglia cells following treatment with E3 or E4 fragments.

RNA transcriptome analyses in BV2 microglia cells following sublethal treatment with nApoE4_1–151_ indicated an upregulation of almost 4,000 genes with 20 of these genes upregulated 182-715-fold as compared to untreated control cells. The majority of these 20 genes play a role in the immune response and polarization toward the microglial M1 activation phenotype. For example several studies have supported that a M1 phenotype is characterized by the upregulation of proinflammatory pathways including TNFα, IL-12, IL-1B, CCl2, whereas a shift to the M2 antinflammatory phenotype leads to the downregulation of genes such as CD86 and CD163 (Shin et al., 2014; Gensel and Zhang, 2015; Yang et al., 2016; Li et al., 2019). Treatment of BV2 cells with nApoE4_1–151_ led to a similar pattern of gene regulation supporting the conclusion of a conversion to a M1 phenotype. Recently, a strong case has been made for ApoE as a pivotal regulator for microglia phenotypes (Krasemann et al., 2017). In this regard, the authors identify a subset termed microglia neurodegenerative phenotype (MGnD) that is characterized by suppressed microglial homeostatic genes such as *P2ry12, Tmem119, Olfml3, Csf1r, Rhob, Cx3cr1, Tgfb1, Mef2a, Mafb, Sall1* and upregulated inflammatory molecules included *Spp1, Itgax, Axl, Lilrb4, Clec7a, Csf1*, and *Apoe*, The authors concluded that as disease progresses, the gene expression profile of microglia switches to MGnD type. We examined the differential gene expression following treatment of BV2 cells with the nApoE4_1–151_ fragment in the context of this study and found a similar increase in *Axl* (eight-fold), *CCl2* (5.6-fold), *Lilrb4* (4.43-fold), and *Spp1* (two-fold). On the other hand, we found a similar decrease in *Tmem119* (−2-fold), *CSF1r* (−1.11-fold), *Rhob* (−1.8-fold), and *Mafb* (−8.22-fold). Therefore, we believe our data support a similar phenotypic change to MGnD in our model system suggesting that nApoE4_1–151_ may lead to a similar conversion of microglia in the AD brain. In a separate study, a novel type of disease-associated microglia (DAM) was characterized and was associated with beta-amyloid plaques in AD, and is also present in ALS (Keren-Shaul et al., 2017). Their analysis indicated that all of the microglia with a DAM signature expressed CD11c and showed an upregulation of other genes including *CD9*, *Clec7a*, and *CD63* (Keren-Shaul et al., 2017). In the present study, we did not find a similar gene expression profile or other DAM-type changes in BV2 cells treated with nApoE fragments, and therefore, we do not believe nApoE4_1–151_ switches microglia to this phenotype in the AD brain.

In conclusion, the nApoE4_1–151_ fragment is a powerful regulator of gene expression, leading to the upregulation of numerous genes linked to inflammation associated with AD. Therefore, preventing the nuclear localization of nApoE4_1–151_, perhaps through the use of structure-corrector molecules (Chen et al., 2012; Mahley and Huang, 2012) may prevent nuclear localization, blocking transcriptional effects and thus, preventing the subsequent inflammation accorded by microglia in AD (Webers et al., 2020).

## Data Availability Statement

The data discussed in this publication have been deposited in NCBI’s Gene Expression Omnibus and are accessible through GEO Series accession number GSE153454: https://www.ncbi.nlm.nih.gov/geo/query/acc.cgi?acc=GSE153454.

## Author Contributions

TR and TP designed experiments, analyzed the data, helped construct tables and figures, and contributed to writing of the manuscript. MM analyzed the data, constructed figures, and contributed to writing of the manuscript. NI, RD, and ES helped carry out experiments. TS and GC helped analyze the data. All authors contributed to the article and approved the submitted version.

## Conflict of Interest

The authors declare that the research was conducted in the absence of any commercial or financial relationships that could be construed as a potential conflict of interest.

## Footnotes

1 https://www.bioinformatics.babraham.ac.uk/projects/trim_galore/

2 www.bioinformatics.babraham.ac.uk/projects/fastqc/

3 pantherdb.org

4 https://www.ncbi.nlm.nih.gov/geo/query/acc.cgi?acc=GSE153454
